# Potential of a Sunflower Seed By-Product as Animal Fat Replacer in Healthier Frankfurters

**DOI:** 10.3390/foods9040445

**Published:** 2020-04-07

**Authors:** Simona Grasso, Tatiana Pintado, Jara Pérez-Jiménez, Claudia Ruiz-Capillas, Ana Maria Herrero

**Affiliations:** 1 Institute of Food, Nutrition and Health, School of Agriculture, Policy and Development, University of Reading, Reading RG6 6AH, UK; 2 Institute of Food Science, Technology and Nutrition (ICTAN-CSIC), 28040 Madrid, Spain; tatianap@ictan.csic.es (T.P.); jara.perez@ictan.csic.es (J.P.-J.); claudia@ictan.csic.es (C.R.-C.); ana.herrero@ictan.csic.es (A.M.H.)

**Keywords:** by-product valorisation, sunflower meal, healthier meat product, spectroscopy analysis, polyphenol profile

## Abstract

Upcycled defatted sunflower seed flour (SUN), a by-product obtained from sunflower oil extraction, was used as an animal fat replacer to develop healthier frankfurters. For that end, animal fat was replaced (~50%) with water and 2% or 4% of SUN. Nutritional composition, technological, structural and sensorial properties were evaluated. SUN incorporation led to a significant increase in protein, minerals (magnesium, potassium, copper and manganese) and a decrease in fat content (~37% less than control with all animal fat). The incorporation of SUN in frankfurters promoted the presence of phenolic compounds. Increasing SUN addition lead to an increasingly (*p* < 0.05) darker frankfurter colour. Samples with SUN at 4% were firmer than the control according to TPA and sensory analysis results and showed the highest lipid disorder attributed to more lipid interactions in the meat matrix. SUN addition as an animal fat replacer in frankfurters is a feasible strategy to valorise sunflower oil by-products and obtain healthier frankfurters.

## 1. Introduction

Sunflower is the fourth crop in the world for oil production after palm, soybean and rapeseed oil [1]. Sunflower meal is the main by-product of the sunflower oil production, representing up to 36% of the mass of the processed seed [2]. The protein content of sunflower seeds is about 20%, whereas the protein content of sunflower meal ranges from 30% to 50% [3]. In addition to protein, sunflower meal contains other valuable nutrients such as vitamins, minerals and polyphenols [2,4]. For this reason, although sunflower meal is mainly used as animal feed [5], it has potential for human consumption [6].

Sunflower meal has been upcycled into a versatile food-grade defatted sunflower seed powder (SUN) [7] with many potential food applications. Grasso et al. [8] showed that SUN has valuable technological properties, such as high water holding capacity. Total phenolic content and antioxidant capacity and nutritional improvements with the use of SUN in biscuits and muffins have recently been reported [8,9]. Nevertheless, the application of SUN in food from animal origin remains to be explored. The use of SUN in popular and highly consumed gel/emulsion meat products such as frankfurters is of particular interest because the SUN’s reported technological properties and nutritional qualities could benefit these products formulations, obtaining healthier meat derivatives. Indeed, several meat products have been reformulated to be healthier using several strategies and ingredients to replace pork back fat [10,11,12]. Examples of animal fat replacements in frankfurters include the use of several plant-based ingredients (but also animal-derived ones such as collagen have been used), such as rye bran and collagen [13], chia seeds [14], pineapple dietary fibres and water [15]. Other animal fat replacements used in meat matrices are mushrooms in pork sausages [16] and grape seed oil emulsified with gelatin and alginate in meat emulsions [17]. However, to the best of our knowledge, there are no studies available on the incorporation of sunflower by-products into popular meat products such as frankfurters.

In this context, this work examined the potential of SUN application as an animal fat replacer on the reformulation of healthier frankfurters. Nutritional composition, sensory acceptability, technological properties (processing loss, colour, pH and texture) and lipid structural characteristics (using ATR–FTIR spectroscopy) were evaluated.

## 2. Materials and Methods

### 2.1. Materials

Fresh post-rigor pork meat (a mixture of *M. biceps femoris*, *M. semimembranosus*, *M. semitendinosus*, *M. gracilis* and *M. adductor*) (30 kg) (23.7% ± 0.2% protein, 3.89% ± 0.49% fat) and pork back fat (5 kg) (0.87% ± 0.1% protein, 92.99% ± 0.80% fat), both from different animals, were obtained from a local market on different days. The meat was trimmed of visible fat and connective tissue. The meat and pork backfat were minced through a grinder with a 0.6 cm plate (Van Dall SRL., Milano, Italy). Lots of approximately 1000 g for meat and 500 g for fat were vacuum packed, frozen and stored at −20 °C until used.

The SUN, donated by the company Planetarians (Palo Alto, CA, USA), was used as an animal fat replacer. This ingredient has been reported to have 38% protein, 1.8% fat and 4.6% moisture, as well as high total phenolic content [8].

Other ingredients and additives used were sodium chloride (Panreac Química, S.A., Barcelona, Spain), sodium tripolyphosphate (Manuel Riesgo, Madrid, Spain), sodium nitrite (Fulka Chemie GmbH, Buchs, Germany) and flavouring (Gewürzmüller, Julio Criado, Madrid, Spain).

### 2.2. Preparation of Frankfurters

Three different types of frankfurter were prepared (Table 1).

One, used as a reference, was formulated with 15% of pork back fat (F/AF) to obtain a final product with about 20% of fat similar to commercial products and other frankfurters [14,15]. The other two samples were elaborated with proximately half of the pork backfat (8%). In these 2 reformulated frankfurters, to replace the rest of pork backfat (7%), SUN was used in 2 levels, 2% (F/2SUN) and 4% (F/4SUN), and the difference was made up with water, according to the formulations shown in Table 1.

Meat and fat were thawed at 3 ± 1 °C prior use. Frankfurters were elaborated according to Jiménez-Colmenero, et al. [18], using the formulation described in Table 1. Briefly, all meat was homogenized and ground for 1 min in a chilled cutter (Stephan Universal Machine UM5, Stephan Söhne GmbH and Co., Hameln, Germany). Then, pork backfat for all samples and the corresponding amount of SUN according to the formulation (Table 1) for each type of frankfurter (F/2SUN or F/4SUN), along with the rest of the ingredients (additives and water) (Table 1) were added to the cutter and mixed for 2 min. Finally, under vacuum and chilled conditions, the mixture was homogenized for 2 min. The final meat batter (<14 °C), was stuffed into 20 mm diameter Nojax cellulose casings (Viscase S.A., Bagnold Cedex, France). Samples were hand-linked and heat processed in a smokehouse (model Unimatic 1000, Micro 40 Eller, Merano, Italy). Then, frankfurters were cooled to room temperature, casings were removed, and samples were vacuum-packed in plastic bags (Cryovac^®^ BB3050, Sealed Air S.L., Sant Boi de Llobregat, Barcelona, Spain) and stored (4 ± 1 °C) until use.

### 2.3. Composition Analysis and Energy Value

#### 2.3.1. Proximate Composition

Moisture and ash contents were evaluated following the Association of Official Analytical Chemists (AOAC) [19] methods. Protein content was measured using a LECO FP-2000 Nitrogen Determinator (Leco Corporation, St Joseph, MI, USA). Fat content was evaluated according to Bligh and Dyer [20]. Fatty acid profiles were carried out in freeze-dried samples (Lyophilizer Telstar Cryodos Equipment, Tarrasa, Barcelona, Spain) by gas chromatography as reported by Pintado, et al. [21] and results were expressed as g of fatty acid per 100 g of product. All the measurements were performed in triplicate.

#### 2.3.2. Dietary Fibre Content

Dietary fibre was determined in SUN and, due to the stability of this food constituent, dietary fibre content in the derived frankfurters was calculated according to the proportion of the ingredient. In particular, dietary fibre was measured by the indigestible fraction method [22], where the sample was subjected to several enzymatic treatments (pepsin, pancreatin, α-amylase, and amyloglucosidase) and dialysis in order to remove the digestible components of the sample and to separate soluble dietary fibre from insoluble dietary fibre. In the soluble dietary fibre, nonstarch polysaccharides were hydrolyzed with sulfuric acid and spectrophotometrically quantitated after alkalinization and reaction with dinitrosalicylic acid [23]. Insoluble dietary fibre was also subjected to hydrolysis, and nonstarch polysaccharides were measured in the supernatant by the same method, while the residue (klason lignin) was determined gravimetrically after overnight drying at 105 °C. Total dietary fibre was determined as the sum of the soluble and the insoluble dietary fibre.

#### 2.3.3. Mineral Content

For mineral content determination, samples were prepared by acid digestion with nitric acid in a microwave digestion system (ETHOS 1, Milestone, Srl, Sorisole, Italy) [24]. The minerals were quantified on a ContrAA 700 High-Resolution Continuum Source spectrophotometer (Analytik Jena AG, Jena, Germany) equipped with a Xenon short-arc lamp (GLE, Berlin, Germany). Three determinations were carried out per sample to measure Ca, Mg, Na, K, P, Fe, Zn, Cu and Mn and results were expressed as mg/100 g product.

#### 2.3.4. Polyphenol Content and Profile

Total extractable polyphenols in SUN and the frankfurters were extracted by means of double aqueous–organic extraction, following the method of Nardoia, et al. [25]. Extractable polyphenols were determined in the corresponding supernatants by the Folin–Ciocalteu procedure [26], using gallic acid (Sigma-Aldrich, St. Louis, MO, USA) as a standard. Results were expressed as gallic acid equivalents (mg GAE/kg of the sample).

Polyphenol profile was additionally evaluated in SUN by HPLC-ESI-QTOF MS analysis in the same extract, after concentration (6:1) with a N_2_ stream, according to procedures previously described [27]. For separation, the HPLC apparatus (Agilent 1200, Agilent Technologies, Santa Clara, CA, USA) was coupled with a diode array detector (DAD) (Agilent G1315B) and a quadrupole time-of-flight (QTOF) mass analyser (Agilent G6530A) with an atmospheric pressure electrospray ionization (ESI). The column used was a 50 mm × 42.1 mm i.d., 3.5 μm, Luna C18 (Phenomenex, Torrance, CA, USA).

Gradient elution was performed with a binary system consisting of 0.1% aqueous formic acid (solvent A) and 0.1% formic acid in acetonitrile (solvent B). The following gradient was applied at a flow rate of 0.4 mL/min: 0 min, 8% B; 10 min, 323% B; 15 min, 50% B; 20 min, 50% B; 23 min, 100% B, followed by a re-equilibration step. The injection volume was 10 μL, and the column temperature was 25 °C. Data were acquired using negative ion mode with a mass range of 100−1200 Da and using a source temperature of 325 °C and a gas flow of 10 L/h. Peak identity was established by comparison with the retention times of commercial standards when available. In addition, the molecular formula proposed by the MassHunter Workstation software version 4.0 for the different signals obtained in the MS experiments were compared with previously reported phenolic compounds, especially in sunflower, and a maximum error of 10 ppm was accepted.

#### 2.3.5. Energy Content

The energy content was calculated based on 9 kcal/g for fat; 4 kcal/g for protein and carbohydrates and 2 kcal/g for dietary fibre [28].

### 2.4. Composition Analysis and Energy Value

#### 2.4.1. Processing Loss and pH

Processing loss was calculated in 10 frankfurters, as the weight loss (expressed as a percentage of initial sample weight) occurring after heat processing and chilling overnight at 2 °C. pH values were determined using an 827 Metrohm pH-meter (Metrohm AG, Zofingen, Switzerland) on homogenates of frankfurters in distilled water in a ratio of 1:10 *w*/*v*. For each sample, 2 homogenates were prepared on which 3 measurements were performed.

#### 2.4.2. Colour

Surface colour determination (CIE-LAB tristimulus values, lightness, L*; redness, a* and yellowness, b*) was evaluated (Konica Minolta CM-3500d colorimeter. Konica Minolta, Madrid, Spain) on cross-sections of the samples. For each type of frankfurter, 10 determinations were carried out.

#### 2.4.3. Texture Analysis

Textural properties were analysed by texture profile analysis (TPA) performed in a TA-XT.plus Texture Analyzer (Texture Technologies Corp., Scarsdale, NY, USA) at approximately 22 °C as described by Bourne [29]. Five cores (height = 20 mm) of each sample were axially compressed to 40% of their original height. Force–time deformation curves were obtained with a 5 kg load cell, applied at a crosshead speed of 1 mm/s. Samples were axially compressed with an aluminium cylinder probe P/32. Attributes were calculated with Texture Expert program as follows: Hardness (Hd) = peak force (N) required for first compression; cohesiveness (Ch) = ratio of active work done under the second compression curve to that done under the first compression curve (dimensionless); springiness (Sp) = distance (mm) the sample recovers after the first compression and chewiness (Cw) = Hd × Ch × Sp (N × mm).

### 2.5. Attenuated Total Reflectance (ATR)-FTIR Spectroscopy Analysis

The infrared spectra of each sample were recorded using a Perkin-Elmer SpectrumTM 400 spectrometer (Perkin Elmer Inc., Tres Cantos, Madrid, Spain) in mid-IR mode, equipped with an attenuated total reflectance (ATR) sampling device containing a diamond/ZnSe crystal according to Pintado, Herrero, Ruiz-Capillas, Triki, Carmona and Jimenez-Colmenero [24]. Measurements were performed at room temperature using approximately 25 mg of the samples (without any previous sample preparation), which were placed on the surface of the ATR crystal, and lightly pressed with a flat-tip plunger. For each type of frankfurter, 9 measurements were carried out. Sums of 3 spectra (24 accumulations) were performed, and these 3-sum spectra were analysed for each sample.

Spectra were acquired with the Spectrum software version 6.3.2 and spectral data were treated with the Grams/AI version 9.1 software (Thermo Electron Corporation, Waltham, MA, USA). The spectral region 3000–2800 cm^−1^ was analysed to study the lipid structural characteristics of the samples [24].

### 2.6. Preliminary Sensory Evaluation

The aim of the sensory test carried out was to have a preliminary evaluation of the products. In this pilot sensory study, frankfurters were assessed by a 27-member consumer panel who regularly consume similar products. Consumers were recruited at random (not targeting specific demographic groups), using mailing lists and were not compensated for taking part. Samples (2.5 cm long) from each formulation were heated for 15 s in a microwave and presented to panellists under green light to minimize colour differences between samples. Participants were instructed to evaluate the following attributes on unstructured 10 cm scales with anchored extremes: Aroma intensity (not to very), firm (not to very), juicy (not to very), powdery (not to very), flavour intensity (not to very) and overall acceptability (dislike extremely to like extremely). Each point was later converted to a numerical scale, 0 for not (aroma intensity, firm, juicy, powdery and flavour intensity) and dislike extremely (overall acceptability) and 10 for very (aroma intensity, firm, juicy, powdery and flavour intensity) and like extremely (overall acceptability). Sensory analysis was performed 2 days after frankfurters’ manufacture.

### 2.7. Statistical Analysis

The frankfurter manufacture was repeated twice on 2 different days. One-way analysis of variance (ANOVA) was performed to evaluate differences between formulations using the SPSS program (v.22, IBM SPSS Inc., Chicago, IL, USA). Least squares differences were used for comparison of mean values between formulations and Tukey’s HSD test to identify significant differences (*p* < 0.05) between formulations.

## 3. Results and Discussion

### 3.1. Composition and Energy Value

Table 2 shows the composition (proximate analysis, mineral content and total extractable polyphenols) and energy value of frankfurters.

**Figure 1:**
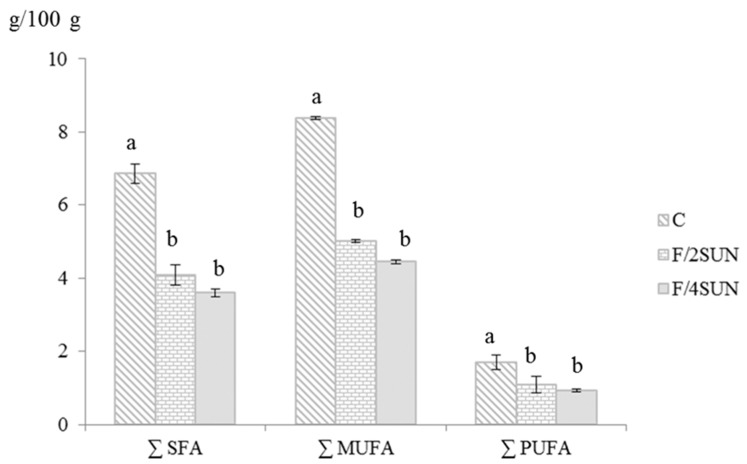
∑ SFA, ∑ MUFA and ∑ PUFA of frankfurters (g/100 g of frankfurter). For sample denomination, see Table 1. Different letters in the same fatty acid group indicate significant differences (*p* < 0.05).

As expected, samples formulated with all pork backfat (F/AF) had the lowest (*p* < 0.05) moisture content, because they were formulated with the lowest water content (Table 1). It was observed that with the SUN addition, protein content significantly increased (Table 2) with increasing SUN addition, due to the protein content of this ingredient [8]. As a consequence of the use of SUN extract as an animal fat replacer, two fat levels were observed, ~19% in samples elaborated with all animal fat (F/AF) and nearer to 12% in frankfurters reformulated with SUN (F/2SUN and F/4SUN) (Table 2). This fat reduction meant a decrease in saturated fatty acids, as can be seen in Figure 1. The unsaturated fatty acids content also was reduced as a consequence of the use of SUN extract as an animal fat replacer in F/2SUN and F/4SUN samples (Figure 1). Overall the SUN addition led to a quantitative fatty acid modification, rather than to a qualitative change in fatty acids.

Regarding dietary fibre, SUN showed a relevant content of 28% in the freeze-dried sample, of which 87% corresponded to insoluble dietary fibre. Since dietary fibre intakes are still far from official recommendations [30], this shows the potential of SUN as a functional ingredient for different food applications. Due to the amount of SUN incorporated into the frankfurters, the final dietary fibre content in F/2SUN was 0.56%, while in F2/4SUN was 1.12%, representing low amounts, although higher than in F/AF, where dietary fibre was absent.

Although absolute values were different for ash content among samples, there was no statistical difference to report (Table 2). Ash content is a good indicator of total mineral content and according to the literature [31], sunflower oil cake on a dry basis contains 0.48% calcium, 0.84% phosphorus, 0.44% magnesium and 3.49% potassium. Not all minerals were analysed in the samples, but the content of some minerals was improved as a consequence of SUN addition in F/2SUN and F/4SUN samples (Table 2). Both F/2SUN and F/4SUN showed higher (*p* < 0.05) magnesium, potassium, copper and manganese concentration than reference samples (F/AF). Regarding zinc, F/2SUN and F/AF showed similar (*p* > 0.05) levels, while F/4SUN had significantly higher amounts. Iron levels increased as a consequence of SUN addition, but the increase was significant only in F/4SUN compared to the control; this was probably due to the fact that most of the iron came from the meat materials and, therefore, only the higher SUN inclusion made a significant difference to the iron content.

Energy value in the control samples (F/AF) was ~232 kcal/100 g, while reformulated samples with SUN, F/2SUN and F/4SUN, showed an energy value of proximately 173 and 180 kcal/100 g, respectively (Table 2). In that sense, the reformulation strategy carried out allowed to obtain products with ~25% less of energy value than control one. This difference in energy value was mainly due to the lower fat content (but also, the higher fibre, water and protein contents) of samples with SUN compared to F/AF.

### 3.2. Polyphenol Content and Profile

Total extractable polyphenol content was determined in the analysed samples (Table 2). A value was obtained for the F/AF samples, despite the absence of SUN, since it is known that in matrixes of animal origin several interfering substances may provide a response to the Folin assay, as observed, for instance, in minced fish [32]. Nevertheless, the most relevant fact is that there was a significant increase in polyphenol content when adding SUN to the frankfurters, even at relatively low concentrations such as 2% and 4%. Whether this presence of phenolic compounds would delay the oxidation of frankfurters remains to be elucidated.

Besides, a total of 24 phenolic compounds were identified in SUN (Table 3): 18 phenolic acids, 5 flavonoids and 1 hydroxycoumarin. Phenolic acids, and particularly quinic derivatives of caffeic or ferulic acids, have been previously reported as the main phenolic compounds in sunflower kernel and shell [33] and, specifically, in sunflower meal [34,35]. These acids were detected in this study, together with some additional phenolic acids (hydroxybenzoic acid, vanillic acid, sinapic acid, a quinolactone from caffeic acid, 1,2-disinapoylgentiobiose and the hydroxyphenylpropionic acid 3,4-dihydroxyphenyl-2-oxypropanoic acid), which, as far as we know, had not been reported in sunflower products before. Regarding the other phenolic compounds, some of them are quite ubiquitous in plant foods, such as (+)-gallocatechin while, for instance, 5-hydroxy-4,4′,6-trimethoxyaureone has not been reported in sunflower seeds, but it has in sunflower flowers as well as in different oils; for these reasons, their presence in SUN was plausible.

### 3.3. Overall Nutritional Value: Nutrition and Health Claims

All samples were a “high in protein” and could be labelled with the corresponding nutritional and health claims according to the Regulation (EC) nº 1924/2006 and Regulation (EU) nº 432/2012 [36,37]. Moreover, as a consequence of the use of SUN as an animal fat replacer, other nutrition and health claims can be made. Taking into account the fat content, the claim “reduced fat content” in F/2SUN and F/4SUN can be made [36]. The increased mineral content due to the presence of SUN, makes it possible for the “source of magnesium” claim to be made on F/2SUN and F/4SUN. In addition, several health claims could be attributed to F/2SUN and F/4SUN according to Regulation (EU) nº 432/2012, due to the magnesium presence. Moreover, F/4SUN could be labelled as “source of copper” and the health claims corresponding to its presence. Regarding zinc and potassium, although their presence was generally higher in F/2SUN and F/4SUN (Table 2), all samples could be labelled as “source of zinc” and “source of potassium” and related health claims [36,37]. These results were similar to other studies where pork back fat in frankfurters was replaced with other high protein ingredients such as hydrolysed collagen [38], and chia seeds [14].

### 3.4. Processing Loss, pH and Colour

The processing loss and pH results are shown in Table 4.

There was no significant difference in the processing loss across the three recipes. These results were low and similar to other frankfurters’ studies, which reported ranges of processing loss between 10% and 20% [39,40,41]. The processing loss obtained (Table 4) indicated good stability in terms of fat and water-binding properties of the meat matrix. The pH decreased with increasing SUN content. The SUN addition influenced the colour, with L*, a* and b* values decreasing significantly with increased SUN content, indicating that the frankfurters became darker, less red and less yellow. It is important that future SUN applications are aimed at minimising colour differences in the food matrix to avoid possible consumer’s rejection, as liking of foods with adequate appearance could favour healthier product consumption by consumers [42]. During the process of new product development, the colour of foods with SUN could be altered to obtain no noticeable colour difference for consumers. Colour alterations have been reported in other studies where pork back fat in frankfurters was replaced by chia seeds [14] and pineapple fibre [15] as well as in other studies using SUN in baked goods [8,9].

### 3.5. Texture Profile Analysis

The results from the texture profile analysis are shown in Table 4. Results showed that hardness was similar (*p* > 0.05) in frankfurters containing 2% of SUN (F/2SUN) but significantly higher in those with 4% (F/4SUN) as compared with the control (F/AF) (Table 4). This is probably due to the water holding capacity of the SUN [8]. The SUN might have retained water, swollen and consequently increased firmness, similarly to the behaviour reported on frankfurters with collagen [13,38,43]. Cohesiveness, springiness and chewiness were higher (*p* < 0.05) in samples with SUN added (F/2SUN and F/4SUN), regardless of the amount of this ingredient used as animal fat replacer (Table 4). This textural behaviour could be related with the differences in composition (mainly protein and fibre content) in each type of frankfurter due to of addition of SUN as an animal fat replacer, although the muscle protein level was kept constant in the formulations.

### 3.6. Attenuated Total Reflectance (ATR)-FTIR Spectroscopy Analysis

**Figure 2:**
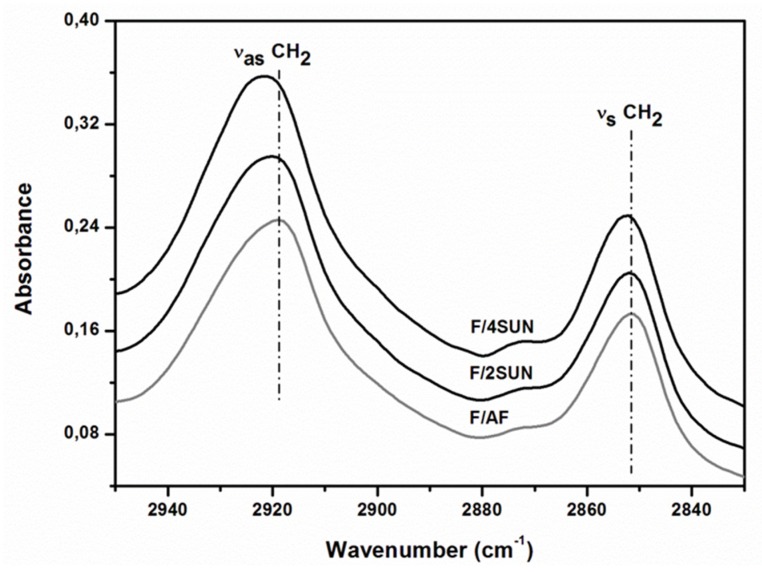
ATR–FTIR spectra in the 2950–2830 cm^−1^ of frankfurters. For sample denomination, see Table 1.

The acyl chain region comprised between 2950–2830 cm^−1^ of the ATR–FTIR spectrum of the different frankfurter are shown in Figure 2.

This spectral region was dominated by two strong bands resulting, respectively, from the asymmetric (_as_CH_2_) and the symmetric (_s_CH_2_) stretching vibrations of the acyl CH_2_ groups [44]. Partial replacement of animal fat by SUN produces a frequency upshift in _as_CH_2_ and _s_CH_2_ from 2919 to 2921 cm^−1^ and from 2851 to 2852 cm^−1^, respectively, in going to from F/AF to F/4SUN (Figure 2). This frequency upshift was generally attributed to the diminution of the conformational order of the lipid acyl chains and to the increase of their dynamics, which implied greater inter- and intramolecular lipid disorder [45,46]. Therefore, it is possible to assume that frankfurters elaborated with SUN showed greater inter- and intramolecular lipid disorder than control elaborated with all animal fat, being these phenomena more relevant in F/4SUN samples. The increase of lipid disorder in reformulated frankfurters as a function of SUN content could be attributed to more lipid interactions in the meat matrix in these samples (F/2SUN and F/4SUN), mainly protein-lipid interactions [47]. The lipid chain disorder or increased lipid interactions observed in F/2SUN and F/4SUN could be related with their specific textural behaviour (Table 3), greater hardness, springiness and chewiness than control (F/AF).

Accordingly, previous studies showed that the direct addition of vegetable proteins, such as soy protein isolate, in the reformulation process of heated meat batters produced protein secondary structural changes accompanied by textural properties modifications such as an increase in hardness and chewiness [48]. In addition, it has been described that the direct addition of mushroom powder, with similar composition as SUN in terms of protein, dietary fibre and bioactive compound content, in the reformulation of processed meats impacts on their rheological and structural characteristics. Similar to results found in the present work, it has been indicated that this reformulation process increased the conformational disorder of the lipid acyl chains due to modifications of lipid-protein interactions [49].

### 3.7. Preliminary Sensory Analysis

The results of the pilot sensory study are shown in Table 5.

The panel detected a significant difference only in the textural sensory parameter’s firmness and juiciness. F/4SUN samples were considered firmer than F/AF, which agrees with findings from the texture profile analysis. F/4SUN samples were also considered significantly less juicy than F/AF. This might be related to another attribute, powdery, which tended to increase in samples with SUN, although not significantly. Samples with SUN tended to have a higher aroma and flavour intensity and lower overall acceptability than control, but these differences were not significant. The reduction of pork back fat in frankfurters has been associated with lower acceptability scores in several sensory attributes [14,16]. Since a limited number of consumers was used to in the present study, sensory tests run on a bigger sample size would need to be repeated in the future in order to ensure enough statistical power.

## 4. Conclusions

The incorporation of SUN as an animal fat replacer in frankfurters offers interesting possibilities for the valorisation of sunflower oil by-products. SUN addition led to several nutritional improvements, including the lipid content (qualitative and quantitative), an increase in protein and minerals. SUN inclusion in frankfurters also promoted the presence of phenolic compounds, mostly phenolic acids. Frankfurters with SUN could benefit from several on-pack nutrition and health claims. Although the reformulation process using SUN as animal fat replacer resulted in some changes in the technological and structural characteristics of the products, the products were found acceptable from a technological point of view. Further sensory tests with a larger consumers’ sample will be needed to build onto the preliminary results from the pilot sensory study here reported. In the future, SUN could also be used as an animal fat alternative in plant-based meat substitutes or cell-cultured meat products as part of an effort to replace animal products and have a positive impact on animal welfare and the environment.

## Figures and Tables

**Table 1:** Formulation (%) of frankfurters.

Samples *	Meat	Pork Back Fat	SUN **	Water
F/AF	61.0	15.0	0.0	21.2
F/2SUN	61.0	8.0	2.0	26.2
F/4SUN	61.0	8.0	4.0	24.2

Additives added to all the samples per 100 g of product: 2 g NaCl; 0.5 g flavouring; 0.3 g sodium tripolyphosphate and 0.012 g sodium nitrite. * Frankfurters formulated with all animal fat (F/AF), half of the animal fat and replacing the rest of fat with 2% (F/2SUN) or 4 % (F/4SUN) of defatted sunflower seed flour (SUN). ** Upcycled defatted sunflower seed flour.

**Table 2:** Proximate composition (%), mineral content (mg/100 g product), total extractable polyphenols (mg/100 g mf) and energy value (kcal/100 g product) of frankfurters.

Parameters	F/AF *	F/2SUN *	F/4SUN *
*Proximate composition*			
Moisture	61.54 ± 0.12 ^c^	64.99 ± 0.19 ^a^	64.47 ± 0.07 ^b^
Protein	15.99 ± 0.04 ^c^	16.96 ± 0.10 ^b^	17.93 ± 0.04 ^a^
Fat	18.67 ± 0.48 ^a^	11.59 ± 0.26 ^b^	11.75 ± 0.10 ^b^
Ash	3.23 ± 0.00 ^a^	2.97 ± 0.18 ^a^	3.36 ± 0.12 ^a^
Dietary fibre **	---	0.56	1.12
*Mineral content*			
Magnesium	36.32 ± 2.73 ^c^	60.72 ± 8.17 ^b^	85.50 ± 3.20 ^a^
Potassium	302.41 ± 10.44 ^c^	383.34 ± 2.98 ^b^	415.16 ± 20.40 ^a^
Iron	1.18 ± 0.09 ^b^	1.57 ± 0.16 ^ab^	2.02 ± 0.18 ^a^
Zinc	2.02 ± 0.04 ^b^	2.28 ± 0.15 ^b^	2.95 ± 0.16 ^a^
Copper	0.06 ± 0.01 ^c^	0.14 ± 0.01 ^b^	0.21 ± 0.01 ^a^
Manganese	0.06 ± 0.00 ^c^	0.18 ± 0.00 ^b^	0.29 ± 0.01 ^a^
*Polyphenols*			
Total extractable polyphenols (mg/100 g mf)	51 ± 9 ^c^	76 ± 11 ^b^	93 ± 12 ^a^
Energy value	231.99	173.21	179.59

* For samples denominations, see Table 1. ** Dietary fibre content was estimated according to the content analysed in SUN, so no statistical analysis was performed. Data are expressed as means ± SD (*n* = 6). Different letters in the same row indicate significant differences (*p* < 0.05).

**Table 3:** Phenolic compounds identified in upcycled defatted sunflower seed flour by HPLC-ESI-QTOF MS analysis.

ID	Class	Subclass	Proposed Compound	Experimental Mass (M-H)^−^	Calculated Mass (M-H)^−^	Error (ppm)	Molecular Formula
1	Phenolic acids	Hydroxybenzoic acids	Hydroxybenzoic acid	137.023	137.0244	10.27	C_7_H_6_O_3_
2	Phenolic acids	Hydroxybenzoic acids	Vanillic acid	167.0344	167.0350	3.47	C_8_H_8_O_4_
3	Phenolic acids	Hydroxycinnamic acids	Caffeic Acid	179.0354	179.0350	2.32	C_9_H_8_O_4_
4	Phenolic acids	Hydroxycinnamic acids	1-O-Caffeoylquinic Acid	353.0864	353.0878	3.27	C_16_H_18_O_9_
5	Phenolic acids	Hydroxycinnamic acids	3-O-Caffeoylquinic acid	353.0915	353.0878	10.43	C_16_H_18_O_9_
6	Phenolic acids	Hydroxycinnamic acids	4-O-Caffeoylquinic acid	353.0849	353.0878	8.21	C_16_H_18_O_9_
7	Phenolic acids	Hydroxycinnamic acids	5-O-Caffeoylquinic acid	353.0899	353.0878	5.91	C_16_H_18_O_9_
8	Phenolic acids	Hydroxycinnamic acids	3,4-di-o-caffeoylquinic acid	515.1230	515.1195	6.78	C_25_H_24_O_12_
9	Phenolic acids	Hydroxycinnamic acids	3,5-di-o-caffeoylquinic acid	515.1200	515.1195	0.97	C_25_H_24_O_12_
10	Phenolic acids	Hydroxycinnamic acids	4,5-di-o-caffeoylquinic acid	515.1211	515.1195	3.10	C_25_H_24_O_12_
11	Phenolic acids	Hydroxycinnamic acids	Caffeoyl-1,5-quinolactone	335.0779	335.0772	1.96	C_16_H_16_O_8_
12	Phenolic acids	Hydroxycinnamic acids	5-O-*p*-Coumaroylquinic acid	337.0949	337.0929	5.94	C_16_H_18_O_8_
13	Phenolic acids	Hydroxycinnamic acids	Feruloylquinic acid	367.1060	367.1035	6.91	C_17_H_20_O_9_
14	Phenolic acids	Hydroxycinnamic acids	Feruloylquinic acid	367.1060	367.1035	6.91	C_17_H_20_O_9_
15	Phenolic acids	Hydroxycinnamic acids	Sinapic acid	223.0605	223.0612	3.11	C_11_H_12_O_5_
16	Phenolic acids	Hydroxycinnamic acids	Hydroxycaffeic acid	195.0315	195.0299	8.18	C_9_H_8_O_5_
17	Phenolic acids	Hydroxycinnamic acids	1,2-Disinapoylgentiobiose	753.2272	753.2248	3.24	C_34_H_42_O_19_
18	Phenolic acids	Hydroxyphenylpropanoic	3,4-dihydroxyphenyl-2-oxypropanoic acid	179.0354	179.0350	2.32	C_9_H_8_O_4_
19	Flavonoids	Aurone flavonoids	5-Hydroxy-4,4′,6-trimethoxyaurone	327.1097	327.1085	0.13	C_18_H_16_O_6_
20	Flavonoids	Dihydroflavonols	Dihydroquercetin 3-O-rhamnoside	449.1089	449.1089	0.08	C_21_H_22_O_11_
21	Flavonoids	Flavanols	(+)-Gallocatechin	305.0695	305.0667	5.96	C_15_H_14_O_7_
22	Flavonoids	Flavonoid glycosides	Didymin	593.1889	593.1876	2.22	C_28_H_34_O_14_
23	Flavonoids	Flavonols	Isorhamnetin hexoside	477.1051	477.1038	2.62	C_22_H_22_O_12_
24	Other polyphenols	Hydroxycoumarins	Esculin	339.0714	339.0722	2.22	C_15_H_16_O_9_

**Table 4:** Processing loss, pH values, colour and texture profile analysis (TPA) of frankfurters.

Parameters	F/AF *	F/2SUN *	F/4SUN *
Processing loss (%)	15.56 ± 1.34 ^a^	16.33 ± 1.10 ^a^	15.46 ± 1.72 ^a^
pH	6.39 ± 0.02 ^a^	6.37 ± 0.05 ^b^	6.34 ± 0.03 ^c^
*Colour parameters*			
L*	76.37 ± 0.50 ^a^	62.33 ± 1.09 ^b^	57.14 ± 0.88 ^c^
a*	6.37 ± 0.27 ^a^	3.24 ± 0.12 ^b^	2.88 ± 0.14 ^c^
b*	11.32 ± 0.31 ^a^	10.21 ± 0.21 ^b^	10.29 ± 0.27 ^b^
*TPA parameters*			
Hardness (N)	17.52 ± 0.92 ^b^	18.27 ± 1.49 ^b^	21.67 ± 2.32 ^a^
Cohesiveness	0.64 ± 0.01 ^b^	0.67 ± 0.01 ^a^	0.67 ± 0.01 ^a^
Springiness (mm)	6.11 ± 0.08 ^b^	6.36 ± 0.08 ^a^	6.41 ± 0.08 ^a^
Chewiness (mm × N)	68.03 ± 3.19 ^c^	77.99 ± 6.06 ^b^	92.35 ± 9.82 ^a^

* For samples denominations, see Table 1. Data are expressed as means ± SD (*n* = 10 for processing loss, *n* = 3 for pH values, *n* = 20 for colour parameters and *n* = 8 for texture parameters). Different letters in the same row indicate significant differences (*p* < 0.05).

**Table 5:** Pilot sensory analysis of frankfurters.

Parameters	F/AF *	F/2SUN *	F/4SUN *
Aroma intensity	3.32 ± 2.34 ^a^	4.19 ± 2.53 ^a^	4.76 ± 2.63 ^a^
Firmness	6.10 ± 1.76 ^b^	6.89 ± 1.51 ^ab^	7.82 ± 2.00 ^a^
Juiciness	5.42 ± 2.29 ^a^	4.70 ± 2.43 ^ab^	3.31 ± 2.34 ^b^
Powdery	1.67 ± 1.51 ^a^	2.53 ± 2.54 ^a^	3.00 ± 2.39 ^a^
Flavour intensity	5.45 ± 2.21 ^a^	5.56 ± 2.15 ^a^	6.54 ± 1.98 ^a^
Overall acceptability	6.07 ± 2.08 ^a^	5.85 ± 2.38 ^a^	4.87 ± 2.60 ^a^

* For samples denominations, see Table 1. Data are expressed as means ± SD (*n* = 27). Different letters in the same row indicate significant differences (*p* < 0.05).
